# An UPLC-MS/MS Method for Simultaneous Quantification of the Components of Shenyanyihao Oral Solution in Rat Plasma

**DOI:** 10.1155/2020/4769267

**Published:** 2020-08-13

**Authors:** Chunbo Jiang, Guoqiang Liang, Yan Ren, Tianjun Xu, Yongliang Song, Weimin Jin

**Affiliations:** ^1^Department of Nephrology, Suzhou Hospital of Traditional Chinese Medicine, Suzhou 215000, China; ^2^Suzhou TCM Hospital Affiliated to Nanjing University of Chinese Medicine, Suzhou 215000, China; ^3^Department of Graduate School, Nanjing University of Chinese Medicine, Nanjing 210023, China

## Abstract

**Objectives:**

To study the quantification of the components in rat plasma after oral administration of Shenyanyihao oral solution.

**Methods:**

Shenyanyihao oral solution has been traditionally used for the treatments of chronic nephritis in clinics. Stachydrine, Danshensu, chlorogenic acid, protocatechuic acid, plantamajoside, aesculetin, isoquercitrin, ferulic acid, baicalin, and baicalein are regarded as the main compounds in Shenyanyihao oral solution. A sensitive, efficient, and precise UPLC-MS/MS method was established and validated for the quantification of the components in rat plasma after oral administration of Shenyanyihao oral solution.

**Results:**

The main pharmacokinetic parameters of the components were acquired based on the analysis of the plasma sample by a noncompartmental method using the WinNonlin7.0 pharmacokinetic program. Danshensu, protocatechuic acid, isoquercitrin, and ferulic acid from Shenyanyihao oral solution were quickly absorbed, and their peak concentration occurred at less than 0.5 h. The pharmacokinetic parameter of the average *t*_1/2_ from Danshensu was 3.91 h in rats, and it was the most rapid distribution and elimination among the components. In addition, the *C*_max_ of stachydrine and baicalin were revealed as the higher plasma concentrations in rats.

**Conclusions:**

This pharmacokinetic study seems to be useful for a further clinical study of Shenyanyihao oral solution in the treatments of chronic nephritis.

## 1. Introduction

Shenyanyihao oral solution, a famous traditional Chinese preparation, was created by experts in traditional Chinese medicine from the school of Wumen based on the pathological characteristics of chronic nephritis, and it is now the internal preparation of the Traditional Chinese Medicine Hospital of Suzhou province, China. It has been used clinically for nearly two decades and is demonstrated to be efficient in improving the clinical symptoms and reducing proteinuria in patients with chronic nephritis [1]. Shenyanyihao oral solution mainly consists of 30 g of *Campanumoea pilosula* Franch, 10 g of *Atractylodes lancea* (Thunb.) DC, 10 g of *Atractylodes macrocephala* Koidz, 30 g of Herba Hedyotidis Diffusae, 30 g of *Pyrrosia lingua* (Thunb.) Farwell, 15 g of *Solanum septemlobum* Bunge, 30 g of *Semen Coix Campanumoea pilosula Franchlacryma-jabi L.* var. *frumentacea* Makino, 20 g of *Scutellaria baicalensis* Georgi, 30 g of *Plantago depressa* Willd., 20 g of *Leonurus japonicus* Houtt., 15 g of polyporus, 10 g of *Angelica sinensis* (Oliv.) Diels, 30 g of *Salvia miltiorrhiza* Bunge, 10 g of *Ligusticum chuanxiong* Hort., and 15 g of *Wolfiporia cocos* [2]. The pharmacological activities of Rhizoma Atractylodis, Rhizoma Atractylodis Macrocephalae, and *Poria cocos*, including strengthening the spleen and expelling dampness, are attributed to their effective constituents [3–5]. Rhizoma Atractylodis, a traditional Chinese medicine, has antibacterial, antiviral, anti-inflammatory, and anticancer activities, and it has been widely used for treating fever, cold, phlegm, and edema in China. Rhizoma Atractylodis Macrocephalae, the dried root of a Compositae plant, has been widely used for its digestive, diuretic, and antihidrotic activities. Previous research demonstrated that Rhizoma Atractylodis Macrocephalae and its compounds could also exert immunoregulation, anti-inflammation, and antidiabetic activities in experimental models [6]. Furthermore, *Hedyotis diffusa*, *Pyrrosia lingua*, and Uncooked Kernels were reported to have the effect of clearing away heat and toxic materials and promoting diuresis [7, 8]. *Hedyotis diffusa*, a traditional Chinese medicine belongs to the Rubiaceae family, has been widely used for the treatment of various inflammation-related diseases, including appendicitis, arthritis, rheumatism, and urethral infection in China. In addition, the activities of Leonurus, Radix Salviae miltiorrhizae, and Angelica primarily focus on improvement of nourishing blood, promoting blood circulation, and resolving dampness [9–20]. A previous study has shown that the combined application of Angelica and Radix Salviae miltiorrhizae could exert the function of activating blood and promoting the production of new blood [12].

Shenyanyihao oral solution is an effective prescription in the treatment of chronic nephritis in China. However, the mechanism and pharmacokinetics of the oral solution in chronic nephritis remain obscure.

In the present study, we first analyzed the material basis of Shenyanyihao and the main components were selected for pharmacokinetic research. Then, a sensitive, efficient, and precise UPLC-MS/MS method was developed to simultaneously determine the analytes and ISs in plasma samples of Shenyanyihao oral solution, including stachydrine, Danshensu, chlorogenic acid, protocatechuic acid, plantamajoside, aesculetin, isoquercitrin, ferulic acid, baicalin, baicalein, carbamazepine, and acetaminophen. The quantitative method was successfully applied to the pharmacokinetic study after oral administration of Shenyanyihao oral solution in rats. The findings would be beneficial for evaluating Shenyanyihao oral solution and exploring the underlying mechanism in the treatment of chronic nephritis.

## 2. Experimental

### 2.1. Chemicals and Reagents

Standards including stachydrine, Danshensu, chlorogenic acid, protocatechuic acid, plantamajoside, aesculetin, isoquercitrin, ferulic acid, baicalin, and baicalein were purchased from Chengdu Biopurify Phytochemicals Ltd. (Chengdu, China). Carbamazepine (IS^+^, purity > 98%) and acetaminophen (IS^−^, purity > 98%) were obtained from the National Institute for Food and Drug Control (Beijing, China). Acetonitrile, methanol, and formic acid (UPLC grade) were from Merck Company (Darmstadt, Germany). Deionized water was purified using a Milli-Q system (Millipore, Milford, MA, USA).

### 2.2. Animals

Male Sprague-Dawley rats (180–220 g) were purchased from Shanghai SLAC Laboratory Animal Co., Ltd. (Shanghai, China) and housed in an environmentally controlled room with a natural light-dark cycle for 7 days before the experiment was carried out. The male Sprague-Dawley rats were randomly given a dose of 10 g/kg/day of Shenyanyihao oral solution by oral administration for pharmacokinetic experiments [13]. All animal experiments were carried out according to the Guidelines for the Care and Use of Laboratory Animals and were approved by the Animal Ethics Committee of the Second Military Medical University.

### 2.3. Preparation of Calibration Standards and QC Samples

Stock solutions of the compounds and the internal standards (ISs) were weighed accurately and dissolved in methanol at 1 mg/ml. The working standard solutions were obtained by mixing the stock solutions of the compounds and then diluting them with acetonitrile to a series of appropriate concentrations. The ISs were mixed and prepared by diluting the stock solutions with acetonitrile to concentrations of 10 ng·ml^−1^ as work solutions. All solutions were stored at 4°C during analysis. Calibration standards were prepared by spiking the working solution into blank rat plasma at different concentrations. Quality control (QC) samples for validation were prepared similar to the calibration standard samples to obtain three different concentrations.

### 2.4. Sample Preparation

The protein precipitation with methanol was applied for sample preparation. 50 *μ*l of plasma spiked with 50 *μ*l of mixed IS solution were thoroughly mixed with 100 *μ*l methanol by vortexing for 30 s. The mixture was centrifuged at 12,000 rpm for 5 min. An aliquot of 100 *μ*l from the supernatant was transferred and subsequently evaporated to dryness by vacuum concentration. Five *μ*l of the supernatant was transferred for the UPLC-MS/MS analysis.

### 2.5. Equipment and LC-MS/MS Conditions

The analytes in plasma were measured by a simple and sensitive UPLC-MS/MS method. Chromatographic analysis was performed on an Agilent 1290 Infinity UPLC system consisting of a binary pump, a surveyor autosampling system, and a thermostatted column compartment. An Agilent Poroshell 120 EC-C_18_ column (3.0 mm × 100 mm, 2.7 *μ*m) was used for chromatographic separation. The mobile phases of A (acetonitrile) and B (0.1% formic acid aqueous solution) were eluted at a flow rate of 0.4 ml/min with the following gradient conditions: 0-5 min, 50% A; 5-6 min, 50%-90% A; and 6-10 min, 90% A. The column temperature was maintained at 25°C, the autosampler was conditioned at 4°C, and the injection volume was 5 *μ*l. The analysis time was 10 min per sample. An Agilent 6470 tandem mass spectrometer (Agilent Technologies, USA) equipped with an Agilent Jet Stream Technology (AJS) electrospray source interface (ESI) was used for MS detection. The mass spectrometric detection was optimized simultaneously in the positive and negative ion modes by multiple reaction monitoring (MRM). The main MS parameters of the ionized chamber are as follows: capillary voltage at 4000 V (positive)/3500 V (negative), gas temperature at 350°C, drying gas flow at 11 l/min, nebulizer pressure at 40 psi, sheath gas temperature at 400°C, and sheath gas flow at 11 l/min. Data acquisition and analysis were performed using the Agilent MassHunter WorkStation version B.07.00.

### 2.6. Method Validation

The analytical method was validated for specificity, linearity, matrix effects, extraction recovery, precision, accuracy, and stability. The specificity of the method was tested by analyzing blank plasma, blank samples spiked with the analytes, and actual samples after oral administration. Plasma samples from rats after oral administration were analyzed for endogenous interference.

Blank plasma was added with the compound stock solutions to prepare a series of calibration standard samples. Calibration curves were generated with peak area ratios of the analytes to IS vs. concentration using 1/*x* weighting. The lower limits of quantitation (LLOQ) were defined as the lowest plasma concentration in the calibration curve.

Replicates of QC samples at three levels were prepared for intraday assay accuracy and precision. The same procedure was performed for 3 consecutive days to determine interday precision and accuracy. The precision was described as relative standard deviation (RSD), and the accuracy was exhibited as relative error (RE).

The matrix effect was determined by comparing the responses of the postextracted standard QC samples with the response of analytes from neat standard samples at three different QC concentrations. The extraction recovery was evaluated by comparing the peak areas of analytes in the extracted plasma samples with those in nonprocessed samples at three different QC concentrations.

The stability was evaluated to cover the anticipated conditions that the samples might be exposed to during storage and handling using QC samples in different conditions. Three levels of QC samples were prepared for analysis under different storage conditions, including short-term stability at room temperature for 3 h, postpreparative stability at the autosampler for 24 h, three freeze-thaw cycles at -80°C, and long-term stability at -80°C for 30 days. Both precision (RSD) and accuracy (RE) should be below 15%.

### 2.7. Pharmacokinetic Study Protocol

The rats were administered orally with Shenyanyihao oral solution. Blood samples from the ophthalmic venous plexus taken at 0.08, 0.17, 0.25, 0.33, 0.5, 1, 1.5, 2, 4, 6, 8, 12, 24, and 48 h after oral administration and at 0 h (predose) were collected (0.2 ml) into heparinized centrifuge tubes. The blood samples were immediately centrifuged at 4000 rpm at 4°C for 5 min, and the supernatant plasma was transferred into another tube and stored at -80°C until treatment.

### 2.8. Data Analysis

Pharmacokinetic parameters were analyzed by a noncompartmental method using the WinNonlin7.0 pharmacokinetic program (Pharsight Corp., USA). The maximum concentration (*C*_max_) and time to reach the maximum concentration (*T*_max_ ) were measured from values obtained from the concentration-time curve. All results were expressed as mean ± standard deviation (SD).

## 3. Results and Discussion

### 3.1. Method Development and Material Basis of Shenyanyihao Oral Solution

Pure acetonitrile was utilized as the precipitant for biosamples. A combination of acetonitrile and methanol mixture was used to evaluate the recoveries and matrix effects. The high extraction efficiency was produced by methanol. Different mobile phases were evaluated to improve LC separation and enhance mass sensitivity of analytes. Modifiers such as formic acid and ammonium acetate were added with different concentrations. The signal intensity of the components was acquired using acetonitrile and formic acid aqueous solution. The components were well isolated under the optimized gradient conditions.

To optimize MS parameters, pure compounds in methanol were individually injected into a MS instrument using MS/MS with MRM mode. The [M + H]^+^ and [M − H]^−^ were used as the predominant ions for analytes in the Q1 spectrum for the different ionization modes in each component. MS/MS working parameters such as precursor-to-product ion pair and collision energy were optimized to obtain the highest intensity of deprotonated molecules of the analytes and ISs. There was no endogenous interference in the actual samples under the optimum conditions. Finally, the analytes were successfully separated and their sensitivity was sufficiently enhanced in this study.

**Figure 1:**
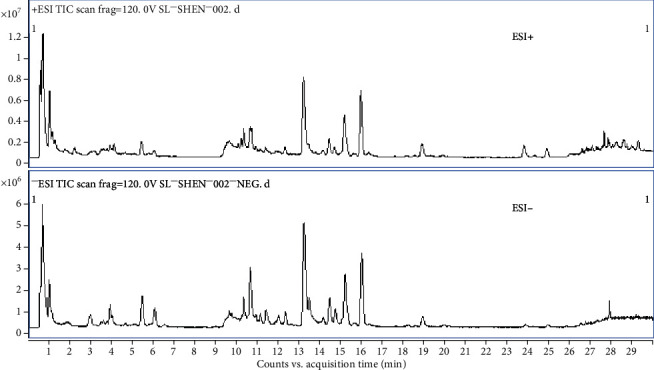
Representative total ion chromatogram of Shenyanyihao oral solution in ESI positive and negative modes.

We conducted a material basis analysis of Shenyanyihao. First, we searched the literature related to the chemical composition of relevant medicinal materials and consulted the information of relevant chemical substances based on the literature reports. Then, we established a database of known chemical compositions through the formula-database-generator software (Agilent Technologies, USA). Next, we searched the total ion current mass spectrum of the medicinal materials in the database, and recorded the retention time, charge mass ratio, and adducted ion of the retrieved chemical components as shown in Figure 1.

We identified compounds from the original data according to the charge mass ratio and confirmed the mass accuracy of these compounds. Elemental composition analysis was conducted using the isotope peak ratio. After database search and verification, the properties of compounds have been initially identified, and the results are shown in Table 1. At last, we selected 10 compounds from them for pharmacokinetic analysis. Structures and characteristic ion peaks of these 10 compounds were shown in Figure S1 and Table 2.

### 3.2. Method Validation

#### 3.2.1. Specificity

**Figure 2:**
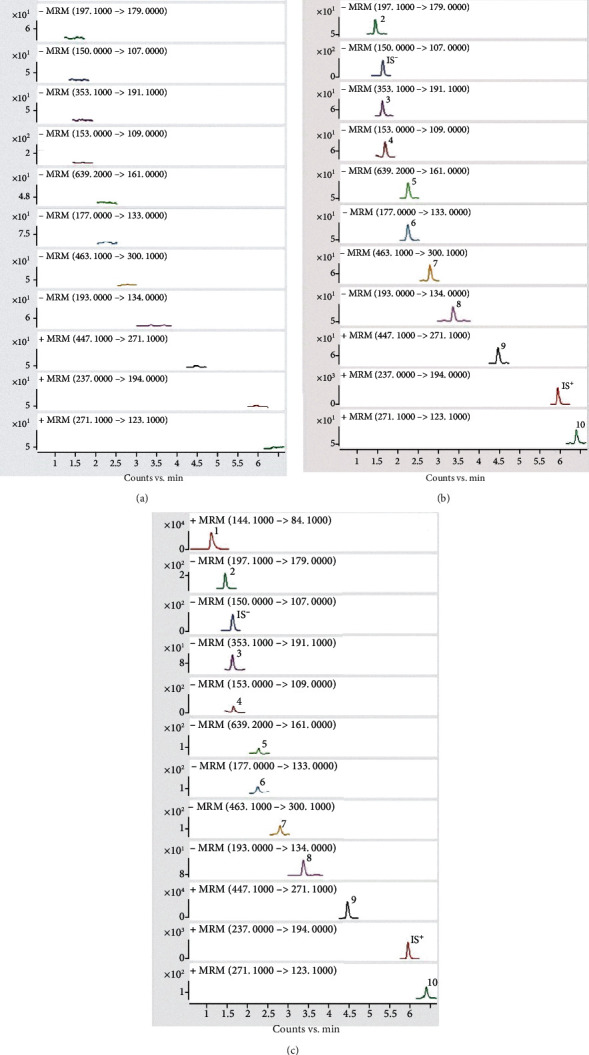
Representative chromatograms of analytes: (a) typical chromatograms of the blank plasma; (b) chromatograms of LLOQ; (c) chromatograms of plasma samples with different components after oral administration. Correspondence between compounds and characters in the graph are as follows: (1) stachydrine, (2) Danshensu, (3) chlorogenic acid, (4) protocatechuic acid, (5) plantamajoside, (6) aesculetin, (7) isoquercitrin, (8) ferulic acid, (9) baicalin, (10) baicalein, (IS^+^) carbamazepine, and (IS^−^) acetaminophen.

Typical chromatograms of the blank plasma (Figure 2(a)), LLOQ (Figure 2(b)), and plasma samples with different components after oral administration (Figure 2(c)) are shown in Figure 2. The results revealed no significant interference peak around the retention time of the analytes and ISs, which accomplished the guideline of bioanalytical method validation.

#### 3.2.2. Calibration Curve and LLOQ

A linear regression analysis using 1/*x* weighting was used to evaluate the linearity of the calibration curve (*y* = *bx* + *a*). The results demonstrated that the calibration curve of the different components in plasma showed good linearity in the matrix over the concentration ranges (Table 3). The recovery rate, accuracy, and precision in LLOQ are measured as shown in Tables 4 and 5.

#### 3.2.3. Precision and Accuracy

The results for the precision and accuracy are shown in Table 4. The intraday and interday accuracy were within -9.60% to 11.60%, while the intraday and interday precision were less than 10.31%. The results showed that the precision and accuracy were within the acceptable range of analysis.

#### 3.2.4. . Extraction Recovery and Matrix Effect

Extraction recovery for all the components and ISs were beyond 82.34% with no significant differences among the three concentrations. In addition, the matrix effect of the analytes ranged from 84.47% to 108.22, which suggested that the method was reliable and no matrix effect occurred (Table 5).

#### 3.2.5. Stability

The stability was determined under different conditions. The results showed that all the components were stable in the plasma of rats at room temperature for 3 h, at 4°C in the autosampler for 24 h, after three freeze-thaw cycles, and at -80°C in a long-term freezer for 30 days (Table 6). In addition, the results showed no significant degradation of analytes under these conditions.

### 3.3. Pharmacokinetic Study

**Figure 3:**
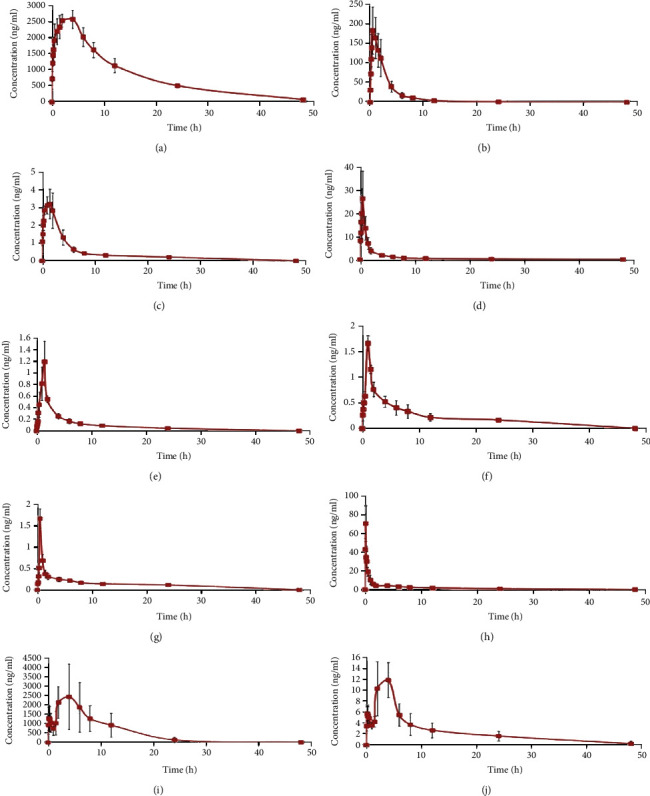
Mean plasma concentration-time curves of analytes after oral administration of Shenyanyihao oral solution: (a) stachydrine; (b) Danshensu; (c) chlorogenic acid; (d) protocatechuic acid; (e) plantamajoside; (f) aesculetin; (g) isoquercitrin; (h) ferulic acid; (i) baicalin; (j) baicalein.

The validated method for the quantitation of the different components was employed to evaluate the pharmacokinetic behaviours in rat plasma after oral administration of Shenyanyihao oral solution. The major pharmacokinetic parameters were evaluated using noncompartmental calculations performed with the WinNonlin7.0 pharmacokinetic program. The plasma concentration-time profiles of all analytes are presented in Figure 3. Among them, the peak concentration of stachydine in Figure 3(a) and baicalin in Figure 3(i) exceeded the upper limit of 2500 ng/ml of the standard curve. So, we measured the point beyond the standard curve by matrix dilution and examined the dilution effect. Dilution integrity was assessed by diluting the samples of high concentration (10 and 50 times concentration of ULOQ) to the quantitative range by blank biological matrix. Five replicates were analyzed for each dilution level. The results of dilution integrity experiments (10x and 50x) suggest that the accuracy was within ±15%, while the precision was under 10%, which conformed to the requirements of the methodology. The major pharmacokinetic parameters are shown in Table 7.

Various drugs from Shenyanyihao oral solution are used together to make up for the treatment of the diseases, including detoxification, dampness, and promotion of blood circulation, which can eliminate the side effects of stubborn dampness heat [14]. Our previous results have confirmed that Shenyanyihao oral solution has a favorable effect on improving the clinical symptoms of the patients with chronic nephritis, which reveals the function of reducing urinary protein, increasing serum albumin, and regulating blood lipids [13]. In addition, we also found that Shenyanyihao oral solution can improve the proteinuria of rats with adriamycin nephropathy and upregulate the expression of nephrin protein in renal tissue. However, the pharmacokinetic study of the components from Shenyanyihao oral solution remains unclear, which sets obstacles on undoing the detailed mechanisms in the treatment of chronic nephritis.

In the present study, the pharmacokinetic parameters of the components in plasma revealed some differences compared with previous researches [16–23]. Danshensu, protocatechuic acid, isoquercitrin, and ferulic acid were quickly absorbed, and their peak concentrations occurred at 0.47 h, 0.50 h, 0.50 h, and 0.17 h, respectively, while stachydrine, baicalin, and baicalein were absorbed much slower than other components for the average *T*_max_  values which were 3.20 h, 2.80 h, and 3.60 h, respectively. Furthermore, the average *t*_1/2_ of Danshensu was 3.91 h in rats, which predicted the most rapid distribution and elimination among the components of Shenyanyihao oral solution. However, the average *t*_1/2_ result of Danshensu was not in accordance with the data from previous reports [17, 24]. The *C*_max_ of stachydrine and baicalin were 2673.0 ng/ml and 2983.4 ng/ml, respectively, and these results were higher than other components of Shenyanyihao oral solution, which denoted higher plasma concentrations of stachydrine and baicalin in rats. Moreover, the AUC_0‐*t*_ and AUC_0‐∞_ values of Danshensu were 556.04 ng/ml∗h and 569.72 ng/ml∗h, and these results were much lower than the data in previous studies [25, 26]. In addition, the *V*_*d*_ values of chlorogenic acid and stachydrine were 1001.03 l/kg and 0.76 l/kg, which exhibited the highest and lowest tissue uptake among the components of Shenyanyihao oral solution after oral administration in rats. The results demonstrated that the different partial pharmacokinetic properties of the components might be related to the metabolic enzyme and interaction system in Shenyanyihao oral solution in rats [27, 28]. The analytical methods and pharmacokinetic parameters could be useful for a deep understanding of the detailed mechanisms of the Shenyanyihao oral solution in the treatment of the chronic nephritis in clinics.

## 4. Conclusion

In conclusion, the present study firstly explored a sensitive, efficient, and precise UPLC-MS/MS method that was developed to simultaneously determine the components and ISs of the Shenyanyihao oral solution in plasma samples. The pharmacokinetic study of the analytes in rat plasma was successfully used by the method after oral administration. The results of the pharmacokinetic parameters were evaluated and analysed to serve as a potential application of the Shenyanyihao oral solution in clinics.

## Figures and Tables

**Table 1:** Compounds identified in Shenyanyihao oral solution.

Identification	RT (min)	Formula	Herbs	Identification	RT (min)	Formula	Herbs
Arginine	0.61	C_6_H_14_N_4_O_2_	Chinese angelica	Isoquercitrin	11.94	C_21_H_20_O_11_	*Hedyotis diffusa*
Stachydrine	0.65	C_7_H_13_NO_2_	Herba leonuri	Dan phenolic acid B	11.99	C_36_H_30_O_16_	The root of red-rooted salvia
Rhamnose	0.65	C_6_H_14_O_6_	*Hedyotis diffusa*	Rosemary acid	12.35	C_18_H_16_O_8_	The root of red-rooted salvia
Glutamate	0.68	C_5_H_9_NO_4_	Chinese angelica	Baicalin	13.22	C_21_H_18_O_11_	*Scutellaria baicalensis*
Aspartic acid	0.69	C_4_H_7_NO_4_	*Hedyotis diffusa*	High front glycosides	14.68	C_22_H_22_O_11_	Plantain herb
Palmitic acid	0.79	C_6_H_5_NO_2_	*Poria cocos*	Digitalis glycosides	14.76	C_31_H_40_O_15_	Plantain herb
Uridine	1.06	C_9_H_12_N_2_O_6_	Agaric polyporus	Red sandalwood of trifoliate bean	15.00	C_22_H_22_O_10_	*Pyrrosia lingua*
Adenosine	1.13	C_10_H_13_N_5_O_4_	Agaric polyporus	Rhubarb phenol	15.13	C_15_H_10_O_4_	Agaric polyporus
Leucine	1.18	C_6_H_13_NO_2_	Chinese angelica	Han baicalin	15.18	C_22_H_20_O_11_	*Scutellaria baicalensis*
Guanosine	1.26	C_10_H_13_N_5_O_5_	Herba leonuri	Wood butterfly	16.28	C_16_H_14_O_5_	*Scutellaria baicalensis*
Phenylalanine	2.22	C_9_H_11_NO_2_	Herba leonuri	Celery	18.24	C_15_H_10_O_5_	Plantain herb
Hydroxymethylfurfural	2.41	C_6_H_6_O_3_	*Codonopsis pilosula*	Hispidulin	18.57	C_16_H_12_O_6_	Plantain herb
Syringic acid	2.96	C_9_H_10_O_5_	Herba leonuri	Salvia diol	21.49	C_18_H_16_O_5_	The root of red-rooted salvia
Danshensu	2.99	C_9_H_10_O_5_	The root of red-rooted salvia	Emodin methyl ether	23.81	C_16_H_12_O_5_	Agaric polyporus
Leonurine	3.18	C_14_H_21_NO_4_	Herba leonuri	2-Hydroxyflavones	23.88	C_15_H_10_O_3_	Agaric polyporus
Hydroxybenzaldehyde	3.22	C_7_H_6_O_2_	Agaric polyporus	Daidzein	24.17	C_15_H_10_O_4_	Agaric polyporus
Vanillic acid	3.22	C_8_H_8_O_4_	Chinese angelica	Methyl rosemary	24.90	C_19_H_18_O_8_	The root of red-rooted salvia
Caffeic acid	4.02	C_9_H_8_O_4_	The root of red-rooted salvia	Emodin methyl ether	24.92	C_16_H_12_O_5_	Agaric polyporus
Tryptophan	4.11	C_11_H_12_N_2_O_2_	Chinese angelica	Hydroxytanshinone	25.53	C_19_H_18_O_4_	The root of red-rooted salvia
Protocatechuic acid	4.54	C_7_H_6_O_3_	The root of red-rooted salvia	Stearic acid	26.88	C_18_H_36_O_2_	Plantain herb
Chlorogenic acid	5.43	C_16_H_18_O_9_	*Pyrrosia lingua*	Implicit tanshinone	26.91	C_19_H_20_O_3_	The root of red-rooted salvia
Aesculetin	6.52	C_9_H_6_O_4_	Chinese angelica	Dihydrotanshinone	27.09	C_18_H_14_O_3_	The root of red-rooted salvia
Rutin	9.39	C_27_H_30_O_16_	*Pyrrosia lingua*	*Salvia miltiorrhiza* new quinone	27.26	C_18_H_16_O_3_	The root of red-rooted salvia
Baicalein	10.10	C_15_H_10_O_6_	*Hedyotis diffusa*	Methyl salvianate	27.37	C_20_H_18_O_5_	The root of red-rooted salvia
Ferulic acid	10.32	C_10_H_10_O_4_	Chinese angelica	Tanshinone	27.64	C_18_H_12_O_3_	The root of red-rooted salvia
Scutellar	10.67	C_21_H_18_O_12_	*Scutellaria baicalensis*	Isocryptotanshinone	27.65	C_19_H_20_O_3_	The root of red-rooted salvia
Purple oxalic acid B	11.35	C_27_H_22_O_12_	The root of red-rooted salvia	Phthalic anhydride	27.87	C_8_H_4_O_3_	Chinese angelica

**Table 2:** Tandem MS parameters of the selected components and two IS.

Compounds	Precursor ion	Product ion	Fragment (V)	Collision energy (eV)	Dynamic time (min)	Ion mode
Stachydrine	144.10	84.10	100.00	30.00	1.10	Negative
Danshensu	197.10	179.00	100.00	10.00	1.50	Negative
Chlorogenic acid	353.10	191.10	100.00	20.00	1.70	Negative
Protocatechuic acid	153.00	109.00	100.00	10.00	1.70	Negative
Plantamajoside	639.2	161.00	100.00	30.00	2.30	Negative
Aesculetin	177.00	133.00	100.00	20.00	2.30	Negative
Isoquercitrin	463.1	300.10	100.00	30.00	2.80	Negative
Ferulic acid	193.00	134.00	100.00	20.00	3.40	Negative
Baicalin	447.10	271.1	100.00	20.00	4.50	Positive
Baicalein	271.10	123.1	100.00	40.00	6.40	Positive

**Table 3:** Calibration curves and LLOQ of the components in plasma.

Compounds	Calibration curve	Linear range (ng/ml)	Correlation coefficient (*r*)	LLOQ (ng/ml)
Stachydrine	*y* = 0.0028*x* + 0.0007	5-2500	0.999	5.00
Danshensu	*y* = 0.0013*x* − 0.00001	1-500	0.999	1.00
Chlorogenic acid	*y* = 0.0100*x* − 0.0007	0.1-50	0.999	0.10
Protocatechuic acid	*y* = 0.0056*x* + 0.0006	0.1-50	0.999	0.10
Plantamajoside	*y* = 0.0020*x* − 0.0001	0.05-25	0.999	0.05
Aesculetin	*y* = 0.0269*x* + 0.0023	0.1-50	0.999	0.10
Isoquercitrin	*y* = 0.0131*x* − 0.0005	0.1-50	0.999	0.10
Ferulic acid	*y* = 0.0028*x* − 0.0056	1-500	0.999	1.00
Baicalin	*y* = 0.0116*x* − 0.0056	5-2500	0.999	5.00
Baicalein	*y* = 0.00097*x* − 0.0003	1-500	0.999	1.00

**Table 4:** Precision and accuracy of the components in rat plasma sample.

Compounds	Concentration (ng/ml)	Intraday (*n* = 5)		Interday (*n* = 15)	
		Precision (RSD%)	Accuracy (RE%)	Precision (RSD%)	Accuracy (RE%)
Stachydrine	10.00	4.17	-6.93	3.51	-5.79
	250.00	6.16	2.35	5.18	0.19
	2000.00	1.92	2.69	2.36	3.49
Danshensu	2.00	8.51	-1.41	7.74	2.93
	50.00	4.37	10.51	10.31	-0.86
	400.00	0.25	8.54	3.60	3.97
Chlorogenic acid	0.20	5.42	-7.99	5.25	-3.25
	5.00	3.22	9.48	1.84	10.40
	40.00	0.29	9.97	1.56	11.60
Protocatechuic acid	0.20	5.49	2.87	4.43	4.77
	5.00	0.58	5.73	2.60	7.88
	40.00	1.93	10.72	1.75	9.02
Plantamajoside	0.10	3.91	6.67	4.78	4.19
	2.50	0.48	3.24	6.59	-4.58
	20.00	1.99	6.38	4.65	0.70
Aesculetin	0.20	1.84	8.45	5.95	1.74
	5.00	1.04	10.08	2.09	8.26
	40.00	0.84	6.87	2.91	8.75
Isoquercitrin	0.20	2.61	4.58	3.53	4.19
	5.00	1.01	5.03	0.98	5.00
	40.00	0.76	10.59	1.31	9.82
Ferulic acid	2.00	7.84	5.39	6.91	3.39
	50.00	1.23	7.27	1.20	6.40
	400.00	0.42	5.69	1.48	4.06
Baicalin	10.00	4.77	-3.66	4.74	-4.40
	250.00	4.32	-9.60	4.50	-8.16
	2000.00	3.08	-1.17	4.21	-5.10
Baicalein	2.00	3.87	-5.68	5.62	-5.18
	50.00	1.67	-8.81	4.15	-6.67
	400.00	4.07	-4.66	4.54	-4.53

**Table 5:** Extraction recovery and matrix effect of the components in rat plasma sample.

Compounds	Concentration (ng/ml)	Extraction recovery (%)	RSD%	Matrix effect (%)	RSD%
Stachydrine	10.00	87.10 ± 1.17	1.34	103.94 ± 4.22	4.06
	250.00	93.12 ± 1.14	1.22	103.81 ± 6.39	6.15
	2000.00	85.02 ± 2.22	2.61	88.58 ± 1.70	1.92
Danshensu	2.00	84.24 ± 0.68	0.81	85.03 ± 7.26	8.54
	50.00	89.22 ± 4.94	5.54	88.22 ± 3.85	4.37
	400.00	85.51 ± 1.04	1.22	85.98 ± 0.22	0.25
Chlorogenic acid	0.20	88.03 ± 3.66	4.16	91.42 ± 8.00	8.75
	5.00	89.21 ± 3.08	3.45	90.33 ± 2.94	3.26
	40.00	87.72 ± 0.45	0.52	92.34 ± 0.27	0.29
Protocatechuic acid	0.20	86.57 ± 0.81	2.09	85.90 ± 3.10	3.61
	5.00	88.33 ± 2.51	2.84	88.74 ± 0.50	0.57
	40.00	85.28 ± 0.28	0.32	88.10 ± 1.69	1.92
Plantamajoside	0.10	87.25 ± 0.88	1.00	85.52 ± 6.29	7.36
	2.50	83.12 ± 2.47	2.98	89.68 ± 0.44	0.49
	20.00	82.34 ± 1.51	1.83	89.84 ± 1.80	2.00
Aesculetin	0.20	83.80 ± 1.60	1.91	98.30 ± 1.30	1.32
	5.00	87.13 ± 1.93	2.22	91.67 ± 0.94	1.02
	40.00	84.64 ± 1.36	1.61	95.37 ± 0.80	0.84
Isoquercitrin	0.20	87.04 ± 4.16	4.78	87.79 ± 2.81	3.20
	5.00	89.18 ± 2.83	3.18	93.97 ± 0.95	1.01
	40.00	86.62 ± 0.62	0.71	92.37 ± 0.70	0.76
Ferulic acid	2.00	95.70 ± 5.63	5.88	94.87 ± 3.82	4.02
	50.00	93.47 ± 3.56	3.81	99.51 ± 1.18	1.19
	400.00	92.69 ± 0.66	0.71	98.60 ± 0.41	0.42
Baicalin	10.00	95.80 ± 3.07	3.20	103.45 ± 5.20	5.02
	250.00	94.92 ± 3.65	3.84	105.52 ± 4.57	4.33
	2000.00	98.81 ± 2.93	2.96	108.22 ± 3.33	3.08
Baicalein	2.00	82.55 ± 1.71	2.07	87.40 ± 4.05	4.63
	50.00	84.04 ± 3.97	4.72	90.89 ± 1.53	1.68
	400.00	87.01 ± 4.56	5.24	84.47 ± 3.44	4.08

**Table 6:** Stability of the components in rat plasma sample.

Compounds	Concentration (ng/ml)	Room temperature (3 h, 25°C)		Autosampler (24 h, 4°C)		Three freeze/thaw cycles		Long term (30 day, −80°C)	
		Precision (RSD%)	Accuracy (RE%)	Precision (RSD%)	Accuracy (RE%)	Precision (RSD%)	Accuracy (RE%)	Precision (RSD%)	Accuracy (RE%)
Stachydrine	10.00	4.62	-9.23	2.66	-9.94	4.44	-7.37	4.69	-5.94
	250.00	0.81	0.09	4.06	-6.0	2.59	6.17	3.49	2.03
	2000.00	4.49	3.14	3.82	3.07	3.55	5.27	3.37	4.64
Danshensu	2.00	5.29	7.09	5.29	7.09	6.92	-2.70	8.08	6.79
	50.00	10.89	-10.20	10.89	-10.20	1.58	-12.52	5.55	-4.84
	400.00	2.78	4.73	2.78	4.73	0.82	0.91	1.22	2.60
Chlorogenic acid	0.20	6.13	-3.40	1.98	-1.57	5.29	-3.89	4.57	-0.48
	5.00	0.45	11.70	0.80	13.89	4.07	9.84	1.25	10.93
	40.00	0.66	11.51	0.65	14.17	1.20	13.15	0.53	12.59
Protocatechuic acid	0.20	3.69	7.78	1.00	12.84	1.60	1.80	3.87	5.18
	5.00	2.55	10.39	1.99	5.52	1.03	8.49	3.58	8.11
	40.00	0.95	10.48	1.97	8.98	2.29	8.55	0.50	7.28
Plantamajoside	0.10	2.00	9.23	5.21	7.94	2.91	6.68	7.05	4.14
	2.50	1.28	-90.31	5.08	-91.35	2.59	-91.11	3.79	-90.84
	20.00	1.36	5.03	1.94	-0.13%	2.87	-5.26	1.20	-0.43
Aesculetin	0.20	5.16	-1.16	1.51	4.88	3.02	2.69	4.20	-0.62
	5.00	2.41	7.25	0.89	8.31	1.95	9.94	1.79	6.76
	40.00	2.07	11.25	4.89	9.87	1.90	7.39	3.61	7.41
Isoquercitrin	0.20	2.65	3.57	2.55	4.81	2.56	8.27	1.88	0.43
	5.00	0.66	5.18	0.48	5.40	1.58	4.20	0.51	5.56
	40.00	0.54	11.02	2.16	11.09	2.43	10.30	0.63	8.27
Ferulic acid	2.00	3.09	5.81%	11.12	6.79	7.50	4.93	4.72	7.22
	50.00	2.37	4.96	0.67	4.55	1.47	6.41	0.99	5.32
	400.00	0.38	3.57	2.20	4.12	1.39	4.29	0.06	2.22
Baicalin	10.00	1.38	-6.39	4.18	-5.56	5.63	-0.46	4.01	-6.58
	250.00	0.37	-10.18	1.38	-11.90	2.72	-0.02	4.76	-5.18
	2000.00	7.12	-3.62	4.10	-6.54	5.25	-7.42	3.31	-5.38
Baicalein	2.00	6.37	-3.82	2.29	-7.65	1.69	-3.44	2.30	-10.32
	50.00	2.29	-9.87	2.99	-8.08	3.72	-1.39	4.60	-5.23
	400.00	5.15	0.51	4.11	-5.84	3.38	-0.69	3.42	-8.34

**Table 7:** The main pharmacokinetic parameters of the components after oral administration of Shenyanyihao oral solution in rats (*n* = 6, mean ± SD).

Parameters	Stachydrine	Danshensu	Chlorogenic acid	Protocatechuic acid	Plantamajoside	Aesculetin	Isoquercitrin	Ferulic acid	Baicalin	Baicalein
*t*_1/2_ (h)	9.09 ± 0.51	3.91 ± 2.34	16.72 ± 3.32	11.35 ± 2.23	12.33 ± 3.92	14.43 ± 4.17	26.39 ± 3.89	11.84 ± 4.98	5.01 ± 0.52	11.72 ± 4.78
*T*_max_ (h)	3.20 ± 1.10	0.47 ± 0.08	1.30 ± 0.57	0.50 ± 0.00	1.40 ± 0.22	1.00 ± 0.00	0.50 ± 0.00	0.17 ± 0.00	2.80 ± 1.10	3.60 ± 0.89
*C*_max_ (ng/ml)	2673.0 ± 163.0	181.79 ± 59.58	3.53 ± 0.52	26.19 ± 11.64	1.31 ± 0.23	1.65 ± 0.15	1.65 ± 0.23	70.24 ± 19.09	2983.4 ± 1344.3	14.25 ± 2.59
AUC_0‐*t*_ (ng/ml∗h)	39256.8 ± 4031.7	556.04 ± 165.66	17.05 ± 2.86	41.96 ± 11.42	3.99 ± 0.44	8.23 ± 1.70	4.75 ± 0.68	76.39 ± 21.43	26525.1 ± 15523.7	106.77 ± 46.19
AUC_0‐∞_ (ng/ml∗h)	40196.0 ± 4088.2	569.72 ± 165.31	21.95 ± 3.34	45.80 ± 12.60	5.01 ± 0.71	11.64 ± 2.41	8.98 ± 1.33	95.70 ± 25.36	26566.8 ± 15520.4	128.95 ± 53.59
AUMC_0‐∞_ (ng/ml∗h^2^)	515393.7 ± 55002.7	2049.56 ± 1061.35	338.84 ± 99.46	307.02 ± 152.54	66.31 ± 28.63	223.19 ± 97.81	304.35 ± 69.29	1254.35 ± 628.41	229094.2 ± 146621.6	2190.95 ± 1741.10
MRT_0‐∞_ (h)	12.82 ± 0.25	3.56 ± 1.71	15.42 ± 3.31	6.60 ± 2.26	12.76 ± 4.47	18.80 ± 5.01	33.67 ± 3.63	12.50 ± 4.16	8.71 ± 2.00	15.16 ± 5.91
*V*_*d*_ (l/kg)	0.76 ± 0.11	13.67 ± 8.51	1001.03 ± 202.53	24.09 ± 4.86	79.97 ± 18.00	7.31 ± 2.02	127.91 ± 13.82	18.01 ± 5.2	12.89 ± 8.01	131.54 ± 23.79
CL (l/kg/h)	0.06 ± 0.01	2.47 ± 0.86	41.83 ± 6.79	1.52 ± 0.47	4.68 ± 0.77	0.36 ± 0.07	3.40 ± 0.50	1.13 ± 0.39	1.71 ± 0.90	8.72 ± 3.03

## Data Availability

The data used to support the findings of this study are available from the corresponding author upon request.

## Supplementary Materials

Supplementary Materials Fig. S1. Structures and characteristic ion peaks of the 10 selected compounds
