# Supplementary material for: An UPLC-MS/MS Method for Simultaneous Quantification of the Components of Shenyanyihao Oral Solution in Rat Plasma
Source: Biomed Res Int. 2020 Aug 13;2020:4769267. doi: 10.1155/2020/4769267 (PMC7443224; doi:10.1155/2020/4769267)
Supplement: Supplementary Materials — Fig. S1. Structures and characteristic ion peaks of the 10 selected compounds [file 4769267.f1.docx]

**Fig. S1.** Structures and characteristic ion peaks of the 10 selected compounds
